# Evaluation of the Bone Union Occurring Subsequent to C1-2 Fusion Combined With C1 Laminectomy for the Surgical Treatment of Retro-Odontoid Pseudotumor

**DOI:** 10.7759/cureus.63422

**Published:** 2024-06-28

**Authors:** Kosei Ono, Sohei Murata, Mutsumi Matsushita, Yu Shimizu, Yusuke Nakamura, Taisuke Yabe, Hiromu Ito

**Affiliations:** 1 Department of Orthopedic Surgery, Kurashiki Central Hospital, Okayama, JPN

**Keywords:** occipitocervical fusion, rheumatoid arthritis, c1–2 fusion, c1 laminectomy, retro-odontoid pseudotumor

## Abstract

Introduction

Surgical treatments for retro-odontoid pseudotumors (ROPs) include C1 laminectomies and C1-2 and occipitocervical (OC) fusions. When a C1 laminectomy is combined with a C1-2 fusion, concerns arise regarding an increased risk of pseudarthrosis due to decreased bone grafting space. Extension of the fusion area to the OC region may be considered to ensure an adequate bone graft bed. However, this procedure is associated with a risk of complications. Thus, in this study, we investigated the bone fusion and clinical outcomes of C1-2 fusion combined with a C1 laminectomy.

Methods

Between January 2017 and December 2022, seven patients with ROPs who had undergone C1-2 fusion combined with a C1 laminectomy were included in the study. All patients were followed up for >1 year. Bone fusion was evaluated by computed tomography (CT) at one year postoperatively, while implant failure was assessed by radiography at the final follow-up. Clinical evaluations included preoperative and one-year postoperative Japanese Orthopaedic Association (JOA) scores and recovery rates.

Results

This study included five male and two female patients, with an average age of 71.9 years. The average follow-up duration was 3.3 years. The primary anchor choices included the C1 lateral mass screw and the C2 pedicle screw. In one case, the transarticular screw was utilized unilaterally, and in another case, a lamina screw was utilized unilaterally. One year postoperatively, CT revealed bone fusion in three of the seven patients. Fusion occurred at the lateral and median atlantoaxial joints in two cases and one case, respectively. Screw loosening was observed in one case. None of the patients required reoperations. The average JOA recovery rate was 34.6%.

Conclusion

This surgical technique is useful for stabilizing and decompressing the C1-2 region while preserving mobility at the OC joint. However, further long-term follow-up studies are required.

## Introduction

In 1986, Sze et al. [1] first described a retro-odontoid pseudotumor (ROP), which has been extensively documented in subsequent studies. The etiologies of ROP include rheumatoid arthritis (RA) [2,3], dialysis [4], and mechanical stress [1,5]. Surgical tumor excision via the oral approach is a known treatment method but carries the risk of numerous complications [1,5,6]. Consequently, posterior approaches have been considered. Posterior approaches include decompression procedures, such as a C1 laminectomy, in addition to fixation procedures, such as a posterior C1-2 fusion and occipitocervical (OC) fusion. C1 laminectomy is a relatively minimally invasive surgery and is deemed beneficial in older adult patients and cases without instability [7]. However, this method may not be suitable for patients with severe instability and is associated with the risk of postoperative anterior arch fracture [8].

Nevertheless, cases of recurrence are known to occur, and reoperation is necessary only when decompression is performed [9]. Therefore, careful monitoring of disease progression is essential. If instability of C1-2 is observed, posterior C1-2 fusion is conventionally considered [10,11]. Additionally, several studies exist regarding the selection of posterior C1-2 fusion, particularly in cases without instability [12,13]. Subsequent to fixation, the ROP tends to decrease, with favorable postoperative outcomes.

Furthermore, reports exist regarding the selection of OC fusion surgery due to the transverse ligament extending to the foramen magnum [14,15]. OC fusion surgery has the advantage of being easily combined with a C1 laminectomy. Nonetheless, OC fusion surgery is associated with the potential risk of serious complications, such as swallowing difficulties and respiratory problems [16], necessitating careful consideration of the indications thereof. A further disadvantage of combining posterior C1-2 fusion with C1 laminectomy is a reduction in the bone graft bed. Therefore, some have advocated for extending the fusion area to the occiput when combined with a C1 laminectomy.

In our department, in cases of massive ROP wherein the approach of posterior C1-2 fusion is chosen, we additionally perform a C1 laminectomy. Typically, posterior C1-2 fusion surgery involves bone grafts, largely from the iliac bone. Therefore, combining a C1 laminectomy with posterior C1-2 fusion might reduce the area available for bone grafting, potentially increasing the risk of pseudarthrosis. However, we have not found any previous studies regarding these challenges. Consequently, in this study, we aimed to evaluate bone fusion and clinical outcomes subsequent to a combined C1 laminectomy and posterior C1-2 fusion as surgical treatment of ROP.

## Materials and methods

Ethical considerations and study design

This study was approved by the Institutional Review Board (IRB) of our institution. Moreover, this study was conducted in accordance with the principles of the Declaration of Helsinki. Written informed consent was obtained from the patients for the publication of their de-identified data and images.

We retrospectively reviewed the data of seven consecutive patients who had undergone posterior C1-2 fusion combined with a C1 laminectomy at our institution between January 2017 and December 2022. The inclusion criteria were a diagnosis of ROP, fixation of C1-2, undergoing C1 laminectomy, and follow-up for more than one year. The exclusion criteria were diagnoses other than ROP, revision surgery, fixation beyond C1-2, not undergoing C1 laminectomy, and follow-up for less than one year. The collected patient data included age, sex, body mass index (BMI), presence of underlying conditions contributing to ROP, such as RA or undergoing dialysis, and the follow-up period.

Radiographically, we evaluated for the presence of a bony bridge in addition to an implant failure, such as an implant fracture or loosening. Computed tomography (CT) and radiography were performed one year postoperatively and at the final follow-up, respectively. Clinically, we assessed the Japanese Orthopaedic Association (JOA) score preoperatively and one year postoperatively, in addition to the JOA recovery rate [17].

Surgical procedure

The patient was placed in the prone position. The C1 and C2 laminae were exposed. Using a navigation system based on three-dimensional images taken intraoperatively, we inserted screws into C1 and C2. Anchors, including the C1 lateral mass screw (LMS), C2 pedicle screw (PS), laminar screw (LS), and transarticular screw (TAS), were selected based on each case. The C1 lamina was excised. Decortication was performed on the C2 laminae and the remaining C1 lamina to facilitate bone grafting. The choice of bone graft, which included local and iliac bones as well as the demineralized bone matrix (DBM), varied depending on the case. A hard cervical collar was worn by the patients for 12 weeks postoperatively.

## Results

Table 1 presents the demographic data of the seven patients. Of the seven patients included in the study, five and two were male and female, respectively, and the average age was 71.9 years. The average BMI was 23.6. Two patients were diagnosed with RA, with no patients undergoing dialysis. The mean duration of the follow-up period was 3.3 years.

**Table 1:** Demographic data of the patients M: male; F: female; BMI: body mass index; RA: rheumatoid arthritis

Case	Age (years)	Sex	BMI	Diagnosis	Follow-up (years)
1	62	M	25	RA	6
2	66	F	22.7	RA	6
3	76	M	22.5		4
4	87	M	19.4		1
5	74	F	24.1		3
6	79	M	23.8		1.5
7	69	M	27.6		1.5

The surgical procedures are presented in Table 2. In all cases, the fusion area included C1-2. C1 laminectomies were performed for all patients, with simultaneous posterior decompression of the mid-lower cervical spine for three patients. The primary anchor choices included the C1 LMS and C2 PS. In one case, a TAS was utilized unilaterally. In another case, a C2 LS was utilized unilaterally. Iliac bone was used in one case, while local bone was used in six cases, which included three cases with DBM as an adjunct.

**Table 2:** Surgical procedures LS: laminar screw; LMS: lateral mass screw; PS: pedicle screw; TAS: transarticular screw; DBM: demineralized bone matrix

Case	Fusion	Decompression	Anchor	Bone graft
1	C1–2	C1, 3–6	C1 LMS C2 PS	Local bone
2	C1–2	C1	TAS (L)/C1 LMS C2 PS (R)	Iliac bone
3	C1–2	C1	C1 LMS C2 PS	Local bone
4	C1–2	C1, 5–6	C1 LMS C2 PS	Local bone + DBM
5	C1–2	C1	C1 LMS C2 PS	Local bone + DBM
6	C1–2	C1	C1 LMS C2 PS (L)/LS (R)	Local bone + DBM
7	C1–2	C1, 6–7	C1 LMS C2 PS	Local bone

Table 3 presents the postoperative outcomes of each case. The one-year postoperative CT revealed that bone bridging was observed in three out of seven patients. The average JOA recovery rate was 34.6%. In one patient, radiolucent lines were observed around the screws, indicating screw loosening. No major complications were observed, and none of the patients required further surgery.

**Table 3:** Radiographic and clinical outcomes JOA: Japanese Orthopaedic Association Score (preopearative → 1 year postoperative)

Case	Bone union	Implant failure	JOA	JOA recovery rate (%)
1	+		8.5→14.5	70.6
2	+		15→15	0
3			7→13.5	65.0
4	+		7→11.5	45.0
5			10→13	42.9
6		Screw loosening	14→12.5	-50.0
7			9→14.5	68.8

**Figure 1:**
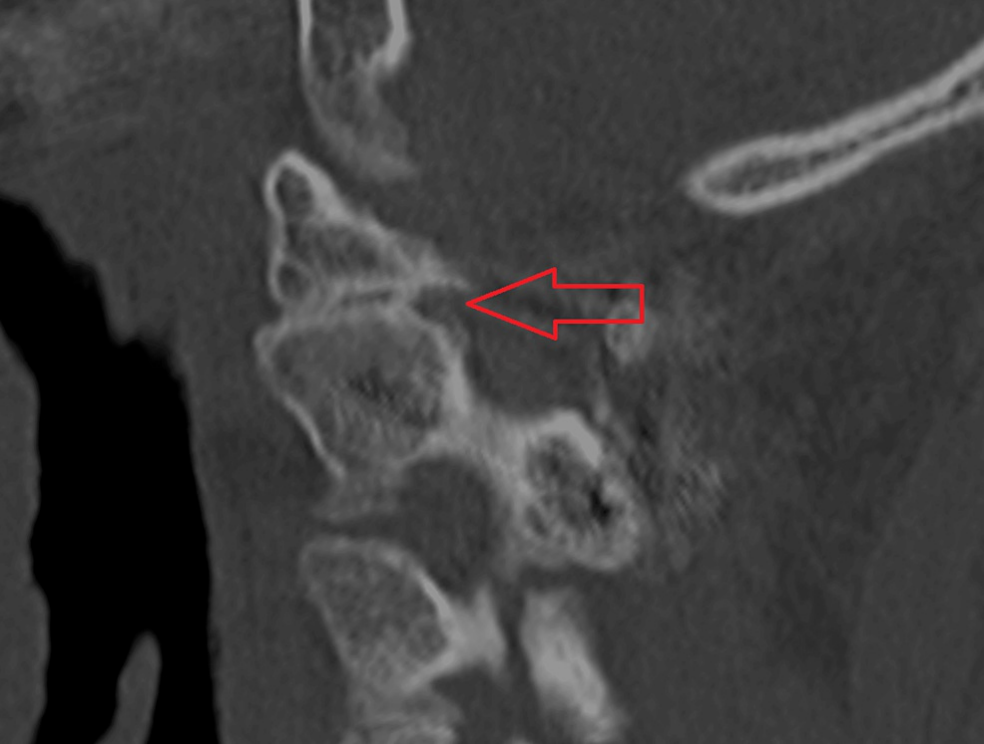
Case 1 Bone union is observed at the lateral atlantoaxial joint.

**Figure 2:**
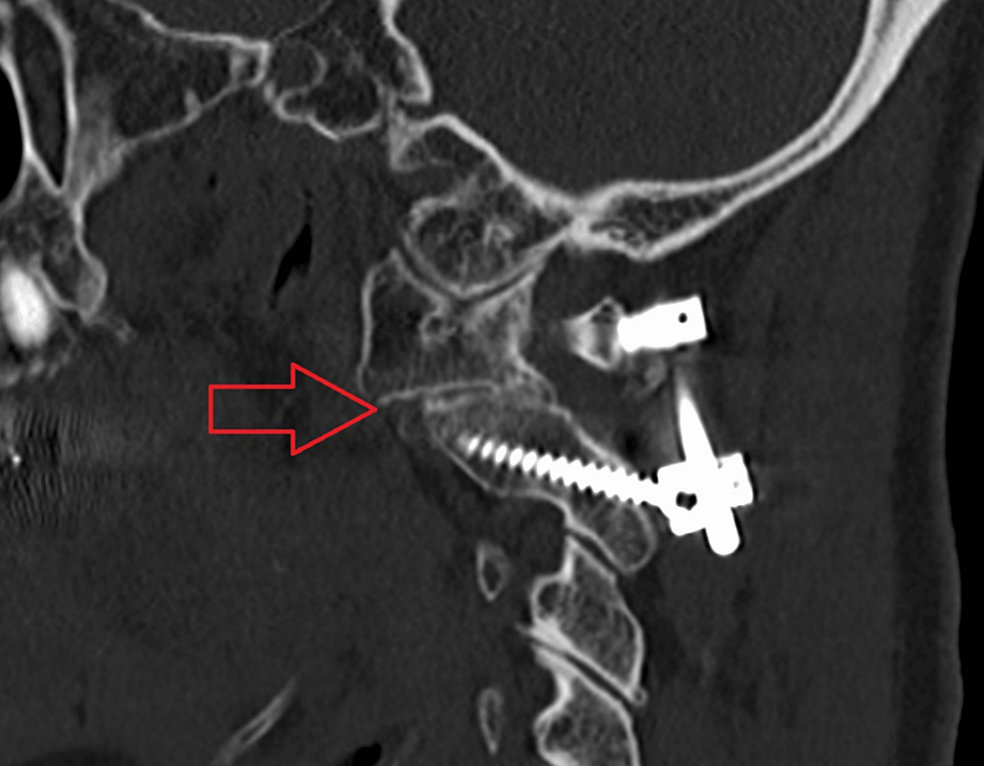
Case 2 Bone union is observed at the lateral atlantoaxial joint.

**Figure 3:**
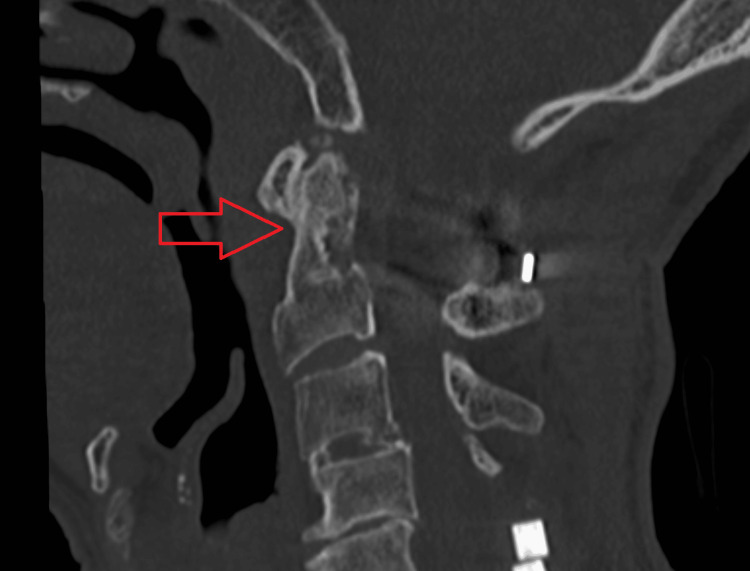
Case 4 Bone union is observed at the median atlantoaxial joint.

We have presented the CT images of three cases in which bone union was achieved. In Cases 1 (Figure 1) and 2 (Figure 2), bone union was observed at the lateral atlantoaxial joints. In Case 4 (Figure 3), bone union was observed at the median atlantoaxial joints.

## Discussion

In this study, we evaluated bone fusion and the clinical outcomes subsequent to a combined C1 laminectomy and posterior C1-2 fusion as surgical treatment for an ROP. Seven patients with ROPs underwent surgical procedures that combined C1-2 fusion and a C1 laminectomy. One year postoperatively, bone fusion was observed in three patients. In cases where bone fusion could not be confirmed, the patients remained clinically stable; none required additional surgery. This surgical technique is useful, allowing for both stabilization and decompression of the C1-2 region while preserving mobility at the OC joint. In our department, in cases of massive ROP, we have combined a C1 laminectomy with posterior C1-2 fusion surgery. Reducing the ROP with fusion alone is time-consuming, and in severe cases, promptly relieving spinal cord compression by removing the C1 lamina proves to be a superior technique.

Using a TAS requires posterior bone grafting, wiring, and tapping for sufficient fixation strength [18], making it unsuitable for use in conjunction with C1 laminectomies. During C1 laminectomies, because bone grafting cannot be adequately performed, the fixation strength of the implants is crucial. Ｗe have primarily selected C1 LMS and C2 PS as anchors. In one case of screw loosening, the C2 LS was utilized unilaterally. We suggest that the use of PS, which provides superior fixation strength [19], would have been preferable.

Various methods are used for bone grafting during C1-2 fixation, including the use of the iliac bone with wiring and tapping. However, bone grafting is challenging in procedures involving C1 laminectomies. An alternative method for bone grafting includes expanding the lateral atlantoaxial joints for bone grafting [20]. However, these joints are covered by venous plexuses, posing the risk of considerable bleeding during the performance of this method. Additionally, the C2 nerve roots are located superior to the lateral atlantoaxial joints and require retraction or detachment, thus risking nerve root injuries.

Therefore, in our department, we do not perform bone grafting on the lateral atlantoaxial joints. As an alternative, we place the bone chips only in the posterior part of the C2 lamina and a small remaining portion of the C1 lamina. Ito et al. [21] have reported on the TAS technique in seven patients. Pseudoarthrosis of the grafted bone occurred postoperatively in all patients, with a bone union at the atlantoaxial joint in five patients. Two patients wherein clear bone union was not observed did not require reoperation. Bone union observed at sites where bone grafting was not performed has been attributed to spontaneous fusion induced by the fixation strength of the implants. Particularly in cases of RA, a natural tendency for joint fusion has been observed, which is thought to have contributed to these results.

In the present study, union at the lateral atlantoaxial joints was revealed in both cases of RA. Statistical analyses were not possible due to the small number of cases. Nonetheless, the potential for bone union in patients with RA suggests that this procedure may be particularly well-suited when compared to patients without RA.

The present study has some limitations. First, this was a retrospective study, which may have had a selection bias. Second, the number of included patients was small. It will be necessary to increase the number of cases for further investigation to accurately detect the rates of bone union, complications, and reoperation. Third, the follow-up period was relatively short; thus, the long-term prognosis was unclear. It is necessary to carefully monitor cases where bone fusion has not been achieved in the CT scan at one year postoperatively to understand their long-term progression. Fourth, other parameters were found to be lacking, such as the visual analog scale and neck disability index, with only the JOA score used as a clinical outcome measure.

## Conclusions

In conclusion, of the seven patients who underwent a C1 laminectomy combined with a posterior C1-2 fusion for the surgical management of ROPs, bone union was observed in three. Clinically stable outcomes were noted, particularly in the four cases in which clear bone union was not observed. Moreover, reoperations did not occur. This procedure proved to be a useful technique that does not require fixation of the occiput; nevertheless, further long-term follow-up studies are necessary.
